# Use of animal-mounted accelerometers to identify positive welfare in dairy cattle

**DOI:** 10.1186/s44363-025-00018-6

**Published:** 2025-10-17

**Authors:** Holly J. Ferguson, Christopher Davison, Joana Lima, Marie J. Haskell, Richard J. Dewhurst, Craig Michie, Ivan Andonovic, Christos Tachtatzis, Ashley Swan, Mark Brooking, Lee Truelove, Laura Shewbridge Carter

**Affiliations:** 1 SRUC, Kings Buildings, Edinburgh, EH9 3JG UK; 2 Department of Electronic and Electrical Engineering, University of Strathclyde, 204 George Street, Glasgow, G1 1XW UK; 3 First Milk, Cirrus House, Glasgow Airport Business Park, Marchburn Drive, Paisley, Scotland, Renfrewshire PA3 2SJ UK

**Keywords:** Dairy cow, Positive welfare, Qualitative Behaviour Assessment, Precision livestock farming

## Abstract

**Background:**

Methods for assessing dairy cattle welfare, such as the gold standard Qualitative Behaviour Assessment (QBA), require training and are time consuming in a sector facing labour constraints and staffing shortages. This research explores whether automated measurements from existing animal-mounted sensors can be used as a basis for objective welfare assessment in dairy cattle, supporting consumer, processor and farmer needs by demonstrating positive welfare on farm. Data (mean standing and lying duration, mean standing and lying bout frequency, mean standing and lying bout duration, mean maximum standing and lying bout duration, mean lying and standing bout frequency, mean step count) were acquired from commercially available ankle-mounted accelerometers intended for oestrus detection and compared to 20 QBA metrics from 107 animals at pasture and during housing on four dairy farms.

**Results:**

Our analysis showed that sensor data was significantly correlated with QBA data, suggesting that sensor data can be used to accurately characterise animal behaviour. Although sensor data was influenced by location (housed and pasture), data obtained from ankle-mounted pedometers showed high accuracy (61%) in predicting an animal’s mood as positive or negative. In addition, step count and standing time were strongly correlated with positive behaviour QBA scores, suggesting that increased step count with decreased standing time may be an indicator of positive welfare. QBA results showed that animals at pasture displayed more positive behaviours, with 70.2% of pasture cattle exhibiting QBA scores associated with positive behaviours/mood, in comparison to 34.0% of housed cattle. Results also showed that skewedness of sensor data from cattle at pasture was an accurate indicator of behavioural synchrony (i.e. animals exhibiting the same behaviour at the same time), a known measure of positive welfare, although more granular time data is needed to investigate further.

**Conclusions:**

This study demonstrates the potential of using automated animal-mounted sensor data to assess positive welfare in dairy cattle, with sensor-derived behavioural features enabling classification of mood states (positive or negative) in 61% of observations.

**Supplementary Information:**

The online version contains supplementary material available at 10.1186/s44363-025-00018-6.

## Background

Dairy cattle welfare is a major concern among both consumers and key industry stakeholders, such as farmers, processors and retailers. Poor cattle welfare can lead to substantial health issues, including lameness, mastitis and infertility, leading to losses in productivity and therefore farm profitability. In addition, consumers have an interest in cattle welfare, with 93% of UK respondents saying they would pay more for products from systems with good cattle welfare [14]. Whilst UK farms must meet standards set by processors, retailers, and farm assurance schemes, the associated welfare assessment methods often focus on absence of a negative indicator, e.g., lameness or dirty cows, as an indicator of the level of welfare. However, absence of negative welfare is not an indication of “positive welfare”.

Positive welfare has been defined as having four distinct features; positive emotions (subjective experiences/affective states), positive affective management (linking positive emotions to goal-directed behaviour), quality of life (balance of negative and positive affective states), and happiness (evaluating the full life of an animal) [29]. Some behavioural trends can be linked to positive welfare. For example, despite lying down being a high priority behaviour for dairy cows [38], longer lying times may not indicate positive welfare and should be considered along with lying bout frequency and duration (number and duration of lying events; see Table 1; [50]). Generally, more frequent short lying bouts are associated with softer lying surfaces [20, 52], whereas less frequent and longer lying bouts can be an indication of lameness [47, 58]. Qualitative Behaviour Assessment (QBA) is the gold standard method to evaluate welfare (Wemelsfelder and Lawrence, 2001), particularly because it can be carried out in both commercial and research settings. It incorporates information on animals’ body language not recorded in previously applied methods [57] and has been included in the Welfare Quality® (WQ protocol [16, 56]. There are two approaches for performing QBA: free choice profiling, where the assessor creates their own behavioural terms (Wemelsfelder et al., 2001), and fixed term, where the assessor uses a list of pre-agreed terms (Keeling et al., 2021).

**Table 1:** Definitions of cow activity data collected via animal-mounted sensor (IceTag, Peacock Technologies, UK)

**Factor**	**Definition**
Mean standing duration (h)	Mean amount of time spent standing up in a 24-h period
Mean standing bout* frequency (count)	Mean number of standing bouts in a 24-h period
Mean standing bout duration (h)	Mean duration of all standing bouts in a 24-h period
Mean maximum standing bout duration (h)	Mean longest standing bout in a 24-h period
Mean lying duration (h)	Mean amount of time spent lying down in a 24-h period
Mean lying bout** frequency (count)	Mean number of lying bouts in a 24-h period
Mean lying bout duration (h)	Mean duration of lying bouts in a 24-h period
Mean maximum lying bout duration (h)	Mean longest lying bout in a 24-h period
Mean step count (count)	Mean number of steps taken in a 24-h period

QBA relies on live human observations and evaluation of animal behaviour, which is a time-consuming task, leading researchers to evaluate alternative and/or automated data collection methods [12, 27]. The use of automated precision livestock farming (PLF) technologies as management tools has the potential to automate data collection for welfare assessment [48, 49], thereby reducing staff time, and improving farm profitability. For example, application of PLF tools in the detection of oestrus or lameness improves herd management, and earlier detection of diseases and disorders improves animal health [30, 41], welfare [3, 7] and production [33]. Although PLF tools are useful for identifying ill-health or a change in animal’s state—and by doing so, can inform on lack of a negative, i.e., fewer cows identified as lame on farm—there is scope for these tools to accurately measure aspects of positive welfare, further improving animals’ health, production, and consequently profitability.

The ability to use technology already on farm to understand more about an animal’s welfare in an automated way would allow farmers a better understanding of their herd health and dynamics. It would also provide a clear increased return on investment from the PLF technologies, with additional benefits reaped from one system. PLF-derived data is verifiable, objective (farmer/processor assessments of welfare may be biased) and non-invasive, reducing any negative impacts that might be associated with animal handling [46]. Although PLF tools are not intended to replace good stockmanship and the skills required to interpret auditory and visual cues [55], they are a useful tool to support farmers in providing the best care and potentially improving accuracy of welfare evaluation.

The aims of this study were to test the possibility of using data automatically gathered from ankle-mounted accelerometers as an indicator of positive welfare, and to assess the accuracy by comparing sensor data with the gold-standard welfare measurement, QBA.

## Methods

Farms were identified for enrolment onto the trial by the industry collaborator (First Milk Ltd.) if they had any existing animal-mounted activity monitoring technology, such as oestrus detection tools. There was no limitation on the type/brand of sensor or data collected and sixteen farms with sensors were enrolled. Of these 16 farms, there were sensors from three companies. However, only one could provide access to sufficiently detailed data to allow further analysis (Peacock Technologies, Edinburgh, UK). Therefore, detailed sensor data from four farms (one in the north of England and three in the South and West of Scotland) were collated and analysed in this feasibility study. All farms had similar dates of turn out to pasture and provided free-stall housing, consisting of bedded mattresses. Surveyed animals were adult, lactating dairy cattle of Holstein, Friesian, or Holstein–Friesian breeds. Activity data included mean lying duration, mean standing time, mean step count, and mean lying and standing bout frequency, duration, and maximum duration, collected via ankle-mounted accelerometers (IceTag, Peacock Technologies, UK; Table 1). Sensor data were collected directly from the sensor provider database. Sensor data were available for analysis for 50 housed animals and 57 pasture animals (*n* = 107). Due to sensor failures, full sensor data were not available for all cows that were behaviourally assessed. Cows with less than 7 days of data during a recording month were excluded from analysis.

QBA was conducted on each farm once during housing periods (Feb 2022) and once during periods at pasture (June 2022) by a single trained researcher to ensure consistency. A total of 20 animals were randomly selected and assessed on each visit. On farms where herds were kept in separate groups when housed or at pasture (e.g. separate high and low yielding animals), animals were randomly selected from each location, to ensure balanced representation of varying conditions. Animals were excluded from the potential pool for QBA if they did not have an IceTag or if they were being treated for poor health, as they were considered not representative of the herd. Lameness was not a criterion for exclusion unless severe.

At each farm, two researchers entered the animal group (indoors or outdoors) immediately following morning milking and walked amongst animals for approximately 20 min, allowing cows to become accustomed to their presence. Following this, an animal was selected at random for assessment. The single assessor watched the chosen cow for 5 min before completing QBA. This was repeated for 20 animals by the single assessor. The QBA assessment consisted of scoring cow behaviour against 20 descriptive terms from the Welfare Quality® protocol (Welfare Quality®, [56]; see supplementary 1). Terms were scored on an unstructured visual analogue scale (VAS) running from minimum to maximum (i.e., a 12.5 cm long line, where ‘minimum’ = the expressive quality indicated by the term is entirely absent and ‘maximum’ = the expressive quality is dominant in observed animals). Following each visit, QBA data was processed, i.e., the length between the minimum point and the assessor’s mark was measured and converted into a score.

To ensure that the number of cows chosen was representative of the overall herd behavioural trends, an analysis of sensor data was conducted. For each farm, a random sample was taken across cows (10, 20, or 30 cows) and days of data per cow (50, 100, or 150 days, split evenly between indoor and outdoor). This process was repeated 30 times, resulting in 270 variations per farm (3 × 3 × 30), except for one farm due to having fewer cows with sensors and less time on farm, which had 150 variations. From each sample, the mean lying time and step count were calculated and compared against the distribution from the full herd to ensure that the sampled period was representative of the overall indoor or outdoor cluster of behaviour for that farm. None of the random samples were distinctly different from the overall herd data during either indoor or outdoor periods.

### Statistical analysis

**Fig. 1:**
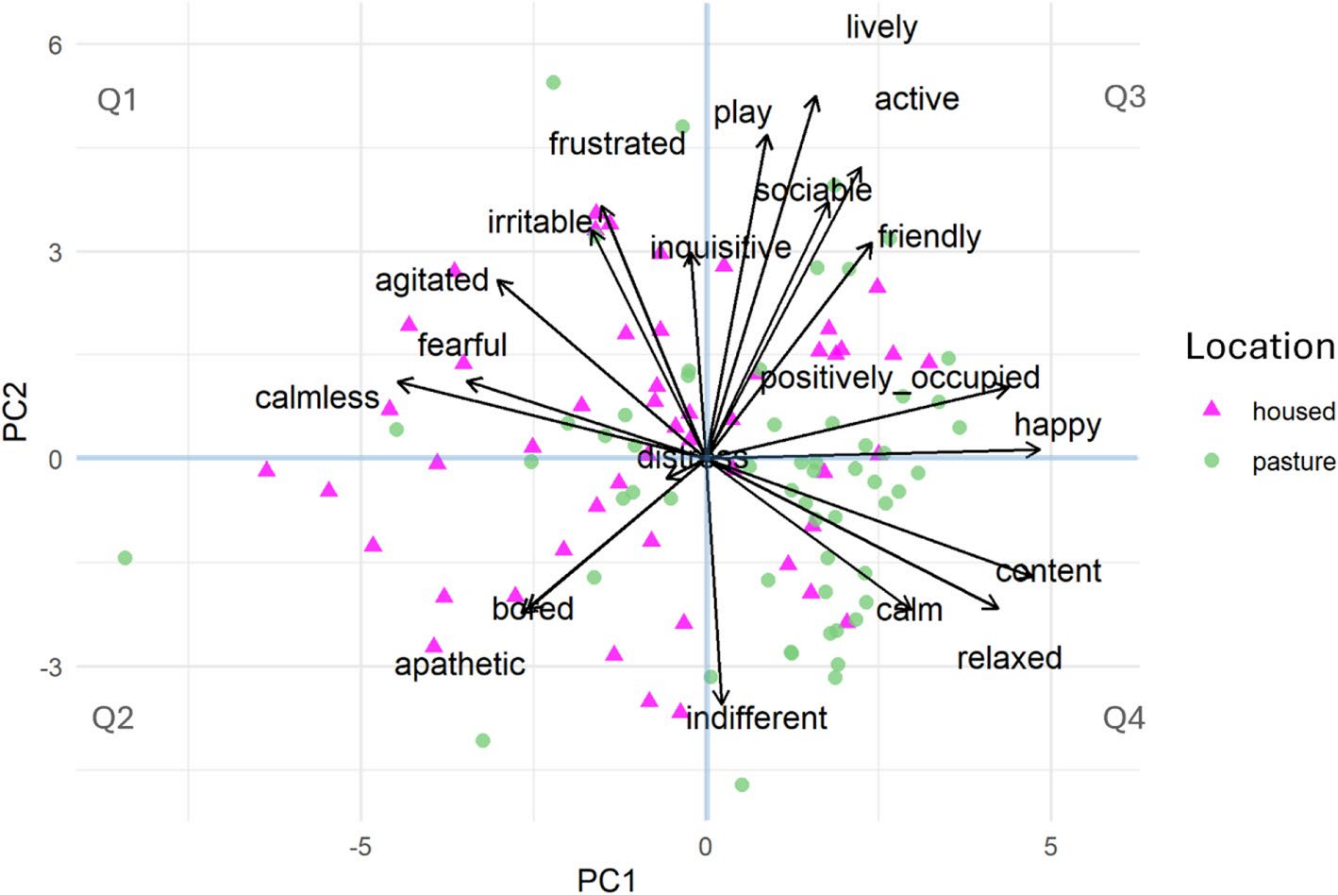
PCA scores plot based on Qualitative Behaviour Asessment (QBA) data, coloured by housing type, with pink triangles and green circles representing data collected when cows were housed and at pasture, respectively (*n* = 107 cows)

Anonymised farm codes were used to ensure researchers were blinded during analysis. To assess and compare clustering patterns between animals, based on the QBA and sensor data, two Principal Component Analysis were conducted (PCA using 2 latent components, using 20 variables for the QBA PCA and 9 variables for the sensor data PCA (mean standing and lying duration, mean standing and lying bout frequency, mean standing and lying bout duration, mean maximum standing and lying bout duration, mean lying and standing bout frequency, mean step count) for 107 cows). The loadings of each variable in each PCA were extracted to evaluate the most important variables driving the special distribution of individual animals. In each PCA, individual animals were assigned to a quadrant (Q), depending on their positioning (top left, bottom left, top right, bottom right as Q1, Q2, Q3 and Q4, respectively). This, as well as an investigation of PCA scores, was performed to allow for comparison of the special distribution of animals in the score plot. (Fig. 1).

Shapiro–Wilk tests were performed to evaluate skewedness as a measure of synchrony, grouped for location (housed or pasture), for each of the 20 QBA terms, as well as QBA-PCA-extracted PCs (QBA-PC1, QBA-PC2), and sensor data and sensor-PCA-extracted PCs (sensor-PC1, sensor-PC2). The QBA terms, QBA-PC1, QBA-PC2, sensor data, sensor-PC1, and sensor PC2 were each analysed using linear mixed-effects models. Farm was included as a random effect (independently distributed with mean 0 and individual variance multiplied by an identity matrix of 4 × 4 order). Location was included as a fixed effect. These analyses were conducted in R software using lme4 [2] and lmerTest [28] packages. All data were analysed using R [42].

To further explore the relationship between the manually collected QBA data and the automatically collected accelerometer data, we calculated the Pearson and Spearman correlations between the behaviour data (20 terms, QBA PC1, QBA PC2) and the sensor data (9 terms, sensor PC1, sensor PC2). Additionally, we computed pairwise distance matrices for both data sets and applied a Mantel test to evaluate the correlation between the behavioural and sensor distance matrices, using Spearman’s rank correlation and 999 permutations to assess statistical significance.

To explore the relationship between the manually collected QBA data and the automatically collected accelerometer data when cows were housed and at pasture, we calculated Spearman correlations between the behaviour data and the sensor data when housed and again at pasture. Significant correlations were defined as *p* < 0.05.

## Results

Comparison of cows during housing and at pasture showed that mean standing duration, mean standing bout duration, mean maximum standing bout duration, mean maximum lying bout duration and mean step count were significantly higher at pasture (Table 2). Mean standing bout frequency, mean lying duration and mean lying bout frequency were significantly higher during housing. Mean lying bout duration was not significantly different between animals during housing and at pasture.

**Table 2:** Summary of results for the effect of location (housed or pasture) on ankle-mounted accelerometer data and principal component (PC) scores obtained from PC analysis of this sensor data for 107 cows. PC1 explained 60.54% variation and was associated with variables that evaluate mood and PC2 explained 24.95% variation and was associated with variables that evaluate activity level

**Factor**	**Housed Mean (± SEM)**	**Pasture Mean (± SEM)**	**t value**	***p* value**
Mean standing duration (h)	12.50 (± 0.34)	13.83 (± 0.22)	3.32	0.001
Mean standing bout frequency (count)	10.51 (± 0.44)	8.24 (± 0.21)	−4.95	< 0.001
Mean standing bout duration (h)	1.38 (± 0.11)	1.78 (± 0.06)	3.25	0.002
Mean maximum standing bout duration (h)	3.90 (± 0.25)	5.30 (± 0.16)	4.81	< 0.001
Mean lying duration (h)	11.50 (± 0.34)	10.17 (± 0.22)	−3.32	0.001
Mean lying bout frequency (count)	10.70 (± 0.46)	8.79 (± 0.22)	−4.00	< 0.001
Mean lying bout duration (h)	1.15 (± 0.04)	1.16 (± 0.02)	0.25	0.81
Mean maximum lying bout duration (h)	2.24 (± 0.07)	2.44 (± 0.04)	2.78	0.01
Mean step count (count)	768.77 (± 35.77)	4218.86 (± 130.20)	24.47	< 0.001
Sensor PC1	−1.16 (± 0.36)	1.02 (± 0.20)	5.44	< 0.001
Sensor PC2	−0.01 (± 0.26)	0.13 (± 0.16)	0.52	0.61

Comparison of QBA data between housing and at pasture showed that positively active, sociable, happy, relaxed, content, and indifferent where significantly higher at pasture (Table 3). Inquisitive, calmless, apathetic, fearful, and playful were significantly higher during housing. There was no difference between lively, irritable, distressed, active, agitated, calm, frustrated, friendly, and bored between housing and pasture.

**Table 3:** Summary of results for the effect of location (housed or pasture) on Qualitative Behaviour Assessment (QBA) data and principal component (PC) scores obtained from PC analysis of these QBA scores for 107 cows from 4 herds. PC1 explained 28.39% variation and was associated with variables that evaluate mood and PC2 explained 19.34% variation and was associated with variables that evaluate activity level

**Factor**	**Housed Mean (± SEM)**	**Pasture Mean (± SEM)**	**t value**	***p* value**
Positively Occupied	8.08 (± 0.34)	10.00 (± 0.34)	3.79	< 0.001
Lively	6.82 (± 0.43)	6.66 (± 0.48)	−0.24	0.81
Inquisitive	5.49 (± 0.44)	3.62 (± 0.38)	−4.26	< 0.001
Irritable	0.72 (± 0.15)	1.05 (± 0.18)	1.37	0.17
Calmless	2.82 (± 0.31)	1.48 (± 0.24)	−3.44	< 0.001
Sociable	7.50 (± 0.45)	9.94 (± 0.25)	5.23	< 0.001
Apathetic	0.47 (± 0.09)	0.28 (± 0.04)	−2.29	0.03
Happy	7.82 (± 0.32)	9.94 (± 0.25)	5.23	< 0.001
Distress	0.44 (± 0.05)	0.42 (± 0.18)	−0.18	0.87
Active	7.20 (± 0.38)	7.85 (± 0.52)	0.99	0.32
Relaxed	8.39 (± 0.28)	9.63 (± 0.30)	3.09	0.003
Fearful	1.35 (± 0.19)	0.73 (± 0.14)	−2.69	0.01
Agitated	1.01 (± 0.16)	0.84 (± 0.13)	−0.81	0.42
Calm	10.27 (± 0.26)	10.73 (± 0.18)	1.67	0.11
Content	8.44 (± 0.28)	10.15 (± 0.28)	4.30	< 0.001
Indifferent	0.71 (± 0.12)	1.14 (± 0.17)	2.01	0.05
Frustrated	0.71 (± 0.12)	0.75 (± 0.20)	0.24	0.81
Friendly	7.30 (± 0.42)	6.68 (± 0.40)	−1.09	0.28
Bored	0.48 (± 0.12)	0.38 (± 0.11)	−0.62	0.53
Playful	2.57 (± 0.42)	1.57 (± 0.34)	−1.98	0.05
QBA PC1	−0.85 (± 0.33)	0.75 (± 0.29)	3.66	< 0.001
QBA PC2	0.25 (± 0.26)	−0.22 (± 0.27)	−1.24	0.22

**Fig. 2:**
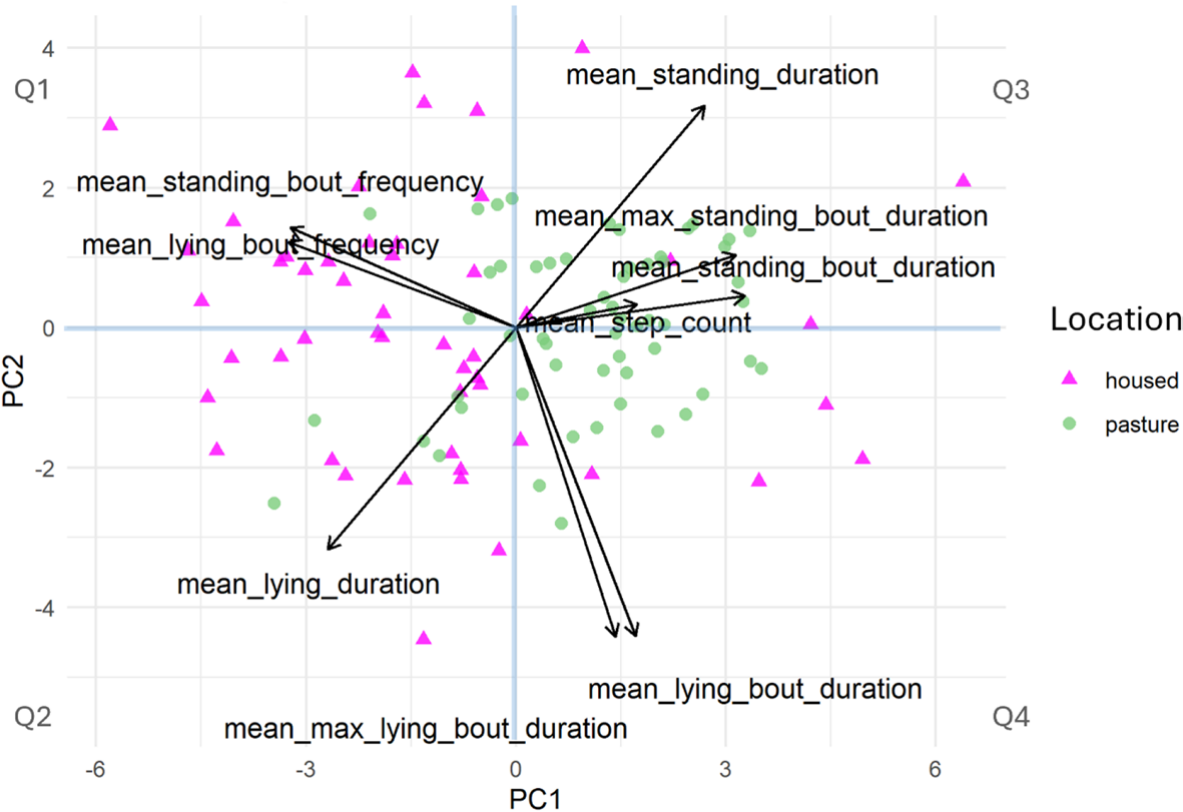
PCA scores plot based on sensor data, coloured by housing type, with pink triangles and green circles representing data collected when cows were housed and at pasture, respectively (*n* = 107 cows)

The PCA based on sensor data showed that PC1 and PC2 explained 60.54% and 24.95%, totalling 85.49% of the variance (Fig. 2). The variables most associated with PC1 (Fig. 1 A) included mean standing bout duration, mean lying bout frequency and mean standing bout frequency, with loadings of 0.41, −0.41 and −0.40, respectively. Regarding PC2, the most important variables were mean lying bout duration, mean maximum lying bout duration, and mean lying duration, with loadings of −0.55, −0.55 and −0.40, respectively (Table 4). Sensor PC1 differed between housed and at pasture, but sensor PC2 did not (Table 2). The Mantel test revealed a significant positive correlation between behavioural and sensor distance matrices (*ρ* = 0.13, *p* = 0.001). Analysing the quadrants of the sensor data-based PCA did not reveal such a clear association between PC1 or PC2 and any defined behaviour, e.g., mean standing duration was similarly important for both PC1 and PC2.

**Table 4:** Principal components analysis loadings based on analysis of data collected from ankle-mounted accelerometer-based sensors from 107 cows from 4 herds

**Variable**	**PC1**	**PC2**
Mean standing duration	0.3368	0.3965
Mean standing bout duration	0.4091	0.0568
Mean maximum standing bout duration	0.3926	0.1296
Mean standing bout frequency	−0.4032	0.1785
Mean lying duration	−0.3368	−0.3965
Mean lying bout duration	0.2139	−0.5511
Mean maximum lying bout duration	0.1782	−0.5522
Mean lying bout frequency	−0.4060	0.1528
Mean step count	0.2161	0.0419

Loadings of principal components 1 and 2 (PC1 and PC2, respectively) were extracted from principal components analysis of sensor data of 107 cows, using 2 latent components

Analysis of the PCA based on QBA data revealed that 28.39% and 19.34% of the variance was explained by PC1 and PC2 respectively, totalling 47.73%. The variables most importantly associated with PC1 included happy, content and calmless, with loadings of 0.37, 0.36 and −0.34, respectively, whereas with PC2 they included lively, playful and active with loadings of 0.40, 0.36 and 0.32, respectively (Table 5). In addition, mood-related variables were more associated with PC1, while activity-related variables were associated with PC2. Mood-related PC1 differed between housed and at pasture, with cows at pasture exhibiting a more positive mood than those housed (Table 3). Activity-related PC2 did no differ between housed and pasture.

**Table 5:** Principal components analysis loadings based on analysis of Qualitative Behaviour Assessment (QBA) from 107 cows from 4 herds

**Variable**	**PC1**	**Variable**	**PC2**
Happy	0.371638	Lively	0.4037
Content	0.362569	Play	0.360531
Positively occupied	0.336708	Active	0.324475
Relaxed	0.325279	Sociable	0.285479
Calm	0.22813	Frustrated	0.281216
Friendly	0.184302	Irritable	0.25454
Active	0.171675	Friendly	0.240291
Sociable	0.135619	Inquisitive	0.229801
Lively	0.121795	Agitated	0.198517
Play	0.067448	Fearful	0.086839
Indifferent	0.017149	Calmless	0.085349
Inquisitive	−0.01742	Positively occupied	0.079772
Distress	−0.04407	Happy	0.010199
Frustrated	−0.11679	Distress	−0.02105
Irritable	−0.12774>	Content	−0.13076
Bored	−0.19915	Relaxed	−0.16572
Apathetic	−0.20567	Bored	−0.16713
Agitated	−0.23284	Calm	−0.16782
Fearful	−0.26694	Apathetic	−0.17172
Calmless	−0.3436	Indifferent	−0.27304

Loadings of principal components 1 and 2 (PC1 and PC2, respectively) were extracted from principal components analysis of Qualitative Behaviour Assessment of 107 cows, using 2 latent components

QBA PC1 score (accounting for 28.39% of total variance) was visually associated with QBA variables that evaluate mood (e.g., fearful, calmless, happy, content), and QBA PC2 score (19.34% of total variance) was associated with QBA variables of activity level (e.g., indifferent, calm, lively, playful).

Spatial distribution of individual animals in PCA plots based on QBA and sensor data, based on the quadrants to which each individual animal had been assigned was analysed. In the QBA-based PCA, animals assigned to Q1 and Q2 (i.e., negative values of PC1) exhibited more behaviours associated with negative mood (e.g. fearful), whereas animals assigned to Q3 and Q4 showed behaviours associated with more positive mood (e.g. happy). In the same QBA-based PCA, animals in Q1 and Q3 were mostly associated with more active behaviours (e.g. lively), and animals in Q2 and Q4 with less active behaviours (e.g. apathetic).

Comparison of spatial distribution of individual animals, based on QBA and sensor data-based PCA was performed via a confusion matrix, based on the quadrants of individual animals in each PCA. This analysis revealed that 61% of the animals in QBA-based Q1 and Q2 remained on Q1 and Q2 when the PCA was based on sensor data. This suggests a moderately high agreement between QBA-based evaluation of animal mood and sensor-based evaluation of animal mood.

Figure 1 shows analysis of the QBA-based PCA score plot coloured by housing type (housed vs pasture), most individual animals located in Q1 and Q2 were sampled while housed, while pasture animals were mostly allocated to Q4.

A total of 70.7% of pasture-based cows exhibited behaviours associated with positive mood (Q3 and Q4), compared to 32.7% of housed cows (*P* < 0.001; Fig. 1). A total of 40% of pasture-based cows exhibited scores associated with active behaviours (Q1 and Q3) compared to 57.1% of housed cows (*P* = 0.22; Fig. 1).

There was a clear distinction between housed and pasture-based animals with regards to their spatial distribution patterns when PCA was based on sensor data (Fig. 2).

To evaluate synchrony of individual herds, and whether synchrony assessed via QBA is similar to synchrony assessed via sensor data, we evaluated the distribution of variables collected from QBA and sensor data, as well as of PC1 and PC2 extracted from both QBA-based and sensor data-based PCAs. Since the distribution of most variables differed due to housing type, these analyses were performed separately.

During housing, QBA-derived PC1 and PC2 scores were normally distributed. The QBA descriptors lively, active, relaxed, and content were also normally distributed. In contrast, descriptors including inquisitive, irritable, calmless, apathetic, distressed, fearful, agitated, indifferent, frustrated, bored, and playful were positively skewed, whereas positively occupied, sociable, happy, calm, and friendly were negatively skewed. Sensor data showed that PC1 was negatively skewed, while PC2 was normally distributed. All sensor-derived variables were normally distributed except for mean standing bout duration and mean maximum standing bout duration, both of which were positively skewed.

At pasture, QBA-derived PC1 scores were negatively skewed, whereas PC2 scores were normally distributed. The distributions of QBA descriptors were generally consistent with those observed during housing, except that *lively*, *active*, *relaxed*, and *content* were negatively skewed, and *sociable* and *friendly* were normally distributed. Sensor data at pasture showed normally distributed PC1 and PC2 scores. Mean step count, mean standing bout duration, mean maximum standing bout duration, mean lying bout duration, and mean maximum lying bout duration were all normally distributed. Mean standing duration was negatively skewed, whereas mean standing bout frequency, mean lying duration, and mean lying bout frequency were positively skewed.

Our results showed that QBA-PC1 reflected mood in cows where QBA-PC2 reflected activity level. Positive behaviours defined by QBA were positively correlated, whereas negative QBA behaviours were negatively correlated with QBA-PC1 (Supplementary 2). In addition, step count (from sensor data) was correlated with QBA-PC1, with step count scaling with QBA-PC1 (Supplementary 2).

Several sensor-derived variables were significantly correlated with QBA measures. For example, step count positively correlated with the QBA term happy whereas mean standing bout frequency and mean standing duration were negatively correlated with happy.

Spearman correlations calculated within location showed that for pasture-based animals, QBA term happy negatively correlated (−0.27) with mean standing bout frequency. Indifferent significantly positively correlated (0.28) with mean maximum lying bout duration. Terms playful (0.39), active (0.30), sociable (0.37), irritable (0.29) and lively (0.29) all positively correlated with mean step count.

For housed animals, QBA term friendly negatively correlated with mean maximum lying bout duration (−0.32). Calm showed positive correlations with mean standing bout frequency (0.30) and mean lying bout frequency (0.29) but negatively correlated with mean maximum standing bout duration (−0.28). Relaxed negatively correlated with mean maximum lying bout duration (−0.30).

## Discussion

The objective of this study was to explore the use of data gathered by animal-mounted accelerometers as a proxy for positive welfare, as assessed via the gold standard welfare assessment method, QBA. Accelerometers are currently deployed on farm to gather data on animal behaviour and activity patterns. Utilising regularly collected data for animal welfare evaluation would further advantage farmers and the industry. In comparison to QBA alone, sensor usage would also allow for earlier detection of health or welfare issues, improving management decisions and enhance overall productivity, without substantially increasing labour demands.

Previous research has shown that QBA scores differ across the day for the same cow (Gutmann et al., 2015) and that housed cows can score better at the start of a housing period compared to the end (de Graaf et al., 2017). To account for this in the present study, QBA was conducted on each farm at a consistent time (after the morning milking). Additionally, farms were only visited for assessment once cows had been housed or turned out to pasture for at least 1 month, ensuring animals were acclimatised to their environments.

Our analysis showed a consistent agreement between QBA and sensor data. We identified that QBA-PC1 and QBA-PC2 were associated with mood and activity levels, respectively. Step count measured via ankle-mounted pedometers and maximum standing bout duration positively correlated with QBA-PC1 score, suggesting that an increased step count, coupled with longer maximum standing bouts are indicative of positive welfare on dairy farms, both for housed cows and cows at pasture. Maximum lying bout duration was negatively correlated with QBA-PC2 score, with lower maximum lying bouts associated with more active behaviours (e.g. agitated, frustrated, playful, lively).

Generally, cows at pasture expressed more positive QBA behaviours compared to housed cattle, indicating a higher level of welfare, in agreement with previous reviews [1, 10], and studies showing cow preference and motivation for accessing pasture [31, 37, 54]. Although not directly comparing activity when housed and at pasture, a study testing the accuracy of ankle-mounted accelerometer sensors (IceQube, Peacocks Technology Ltd.) in different housing conditions, reported an average of 19 steps/h when cows were housed compared to 120 step/h at pasture [9]. Animals in this present study had overall higher step counts, both at pasture and during housing, but followed similar patterns.

There was clear distinction between housed and pasture-based animals with regards to their spatial distribution patterns from PCA based on QBA. Showing more positive behaviours, most pasture-based cattle fell into Q3 and Q4. For housed cattle, spatial distribution for QBA-based PCA showed most individual animals located in Q1 and Q2. Animals in Q1 during housing – showing high activity, low mood – could be associated with various negative aspects of housing and management. For example, animals could score higher on activity and lower on mood with increased competition – limited resources such as cubicle access or feed face access [23], stress – stocking density, slippery floors, presence of assessors could also increase activity levels [23], discomfort or pain – illness can affect lying times, as can uncomfortable or small mattresses [44]. Identification of farms with animals in these negative quadrants via automatically collected sensor data, could highlight scope for improvement,reducing stocking density, access to feed face, improving mattress comfort, improving enrichment and social enrichment opportunities would all increase animal welfare [32].

Step count may be higher for cows at pasture due to behavioural changes, such as grazing behaviour [13], and due to increased walking distances to the milking parlour [40]. An increase in step count at pasture, coupled with the knowledge that cows tend to have improved welfare at pasture, may account for the correlations seen between QBA data associated with positive welfare and step count (from sensor) in this dataset. However, the relationship between welfare, housing and standing time is less clear. For example, increased standing times have been associated with uncomfortable lying surfaces [19], comfortable standing surfaces [18, 22, 53], or thermal stress [21] and decreased standing times associated with increased lameness [5]. In agreement, in this study, we observed a negative relationship between QBA term happy and mean standing bout frequency (−0.27) at pasture. Additionally, mean step count at pasture was positively correlated with lively (0.29), sociable (0.37), active (0.30) and playful (0.39). However, in housed cattle we observed a negative correlation between QBA term calm and mean maximum standing bout duration (−0.28) and between relaxed and mean maximum lying bout duration (−0.30).

This is in agreement with previous work which has shown that cows which are comfortable enough to move freely and without pain, and have comfortable lying surfaces have increased lying bout frequency, i.e. lie for longer times across the day but the duration of each individual bout is shorter, and animals stand up more frequently, though stand for a shorter period of time [11]. Conversely, difficulty rising—from lameness, poor or uncomfortable lying surfaces or bullying cows – is associated with increased standing time and reduced lying bout frequency, e.g., once an animal is standing, she is more likely to remain standing [11].

The combination of increased step count with longer maximum standing bouts observed for housed cattle in this present study, therefore, may be an important indicator of positive welfare. The quality of housing on the farms in this study was of a similar standard and further research on farms with a variety of housing qualities is ongoing to better understand these correlations.

A study using QBA to assess welfare in housed beef cattle compared results from live QBA observations to those carried out on video recordings of the animals for the same time-period [12]. Although there was reasonable agreement between the live and video QBA observations for animal activity level and mood, video QBA results lacked sensitivity with regards to animal mood. Despite the variations observed in sensor data for housed and pasture-based animals in this study, it was still possible to predict an animal’s mood as being positive or negative with 61% accuracy, based on sensor data alone from 4 farms, regardless of location. This suggests that it is possible to use data from sensors already on farm for management purposes to understand more about the level of positive welfare on farm in an automated way. Focusing on mood as positive also allows farmers to move from the outdated “lack of negative” aspects of animal welfare, standard in many assurance schemes. Not only would an automated welfare assessment provide a non-biased understanding of welfare levels on farm, removing any consumer doubt, but utilising technologies for additional aspects of management can reduce labour and time demand for farmers, allowing time to be allocated to other important tasks. Demonstration of additional benefits of PLF tools, such as increased welfare monitoring or reduction of carbon footprint from improved fertility, health, and management [15, 34] could further increase uptake of these technologies across the sector, improving the return on investment in PLF tools for farmers.

There is no globally accepted definition of “animal welfare”. However, the theme of a “natural life” or a life worth living recurs in literature [17, 35], within government [6], and throughout public opinion [8, 24]. Due to successful domestication of cattle, it is difficult to assess their natural behaviours [43]. A review of cattle behaviour in 22 herds with minimal human interference found that behavioural synchrony (performing the same behaviours at the same time) was a feature of semi-natural herds [26]. Thus, behavioural synchrony is often used as an indicator of positive welfare in cattle (Napolitano et al., 2009). In this study, behavioural synchrony at pasture may have influenced the distribution of sensor data and PC scores. At pasture, distributions for QBA-PC1 scores and standing times were skewed compared to housed data – i.e., at pasture more cows displayed more positive QBA behaviours and had more similar standing times. However, for step count, data were skewed when cows were housed compared to pasture, which could suggest an ability to express a greater range of steps at pasture, which may be an indication of positive welfare. Conversely, the lack of variation in step count when housed may be an indication of behavioural constraint. Further research with data of a higher granularity is being investigated as part of a follow-on study, to explore whether these skewed distributions are a true representation of synchronous behaviour at pasture or an artefact of the impact of grazing and distance to parlour.

Lying behaviour is important for cattle, with cows highly motivated to lie down and for a set amount of time per day (Metz, 1985; [25]). Lying behaviour takes priority over feeding and social contact when all three behaviours are deprived [38]. In the current study, housed cows lay down for longer (11.5 h/d) than those at pasture (10.2 h/d), despite having a known high motivation to lie down in open areas [45, 51, 54]. This may be due to cows at pasture spending more time grazing compared to time eating when housed (Roca Fernandez et al., 2013), giving cows the opportunity to lie down for longer when housed. Alternatively, lameness can cause an increase in lying times and suggests that higher lying time can indicate poorer welfare [4, 5]. Therefore, lying time is often reported along with lying bout frequency and lying bout duration for a comprehensive understanding of the lying behaviour [50]. Generally, longer and fewer lying bouts are associated with harder, more uncomfortable lying surfaces, with cows reluctant to get up after lying down [20, 52]. The current study found that cows lay down more often when housed, with lying bout duration being the only sensor-recorded behaviour not to differ between housed and pasture animals in this study. The use of lying behaviour data in the current study to assess positive welfare is inconclusive. Had the housing conditions of these farms been poorer and/or ranked, differences in lying behaviours may have been able to be used as a welfare indicator. Ongoing research involves farms with varying levels of expected welfare and housing quality to assess the impacts this has on QBA data, sensor data and relationships between the two.

There were limitations to this study which were addressed where possible and will be addressed in future research. For example, there was only one commercial company who could provide sufficient data for analysis i.e. not processed in a way which reduced detail. This limited the number of farms included in the present study to four, though this has been rectified in a follow-on study with 15 farms. Inclement weather and management practices could have impacted the QBA scores collected during the study. To try to combat this, farm visits were grouped into weeks, to try to ensure similar weather conditions. If conditions were extremely poor, the visit was rescheduled. Farms were visited following the morning milking on each farm to try to ensure animals were at the same “stage” in their day, regardless of farm variation. If management procedures such as hoof trimming or veterinary visits were to take place, QBA visits were rescheduled.

## Conclusions

Overall, this study demonstrates the potential of leveraging automated animal-mounted sensor data to assess positive welfare in dairy cattle. Sensor-derived features from ankle-mounted accelerometers enabled the classification of affective states (positive vs. negative mood) in 61% of observations for 107 animals across four farms. Pedometers, intended to be applied to evaluate oestrus and lameness, can further farmer knowledge of the welfare of their herds, known to impact productivity and farm profitability, allowing improvements to be made where necessary. Correlations between step count and standing bout durations with QBA data suggest that increased step count, coupled with longer maximum standing bouts may be used as a potential indicator for positive welfare. However, further work should be conducted to provide more robust evidence from a greater number of farms.

Additionally, this work has shown that PLF monitoring systems may also detect patterns associated with behavioural synchrony, a known indicator for positive welfare. This research was conducted as a feasibility study across four farms. Additional data gathering across more farms with diverse expected welfare and a higher granularity of sensor data is being executed in a follow-on study.

The reported research provides evidence to the dairy industry of a potential automated measure of on-farm positive welfare, using technologies widely commercially available on the market and not relying on farmer and processor assessment of welfare alone. This information could be used to inform and gain trust from consumers, as well as being utilised for welfare schemes and by processors.

## Supplementary Information

Supplementary Material 1. 

## Data Availability

The datasets used and/or analysed during the current study are available from the corresponding author on reasonable request.
