# Supplementary material for: Use of animal-mounted accelerometers to identify positive welfare in dairy cattle
Source: Dairy Sci Manag. 2025 Oct 17;2(1):15. doi: 10.1186/s44363-025-00018-6 (PMC12637375; doi:10.1186/s44363-025-00018-6)

Supplementary Material: Use of animal-mounted accelerometers to identify positive welfare in dairy cattle

Ferguson HF, Davison C, Lima J, Haskell MJ, Dewhurst RJ, Michie C, Andonovic I, Tachtatzis C, Stamper A, Brooking M, Truelove L, Shewbridge Carter L

**Supplementary 1:**

*Summarised Welfare Quality Assessment Qualitative Behaviour Analysis (QBA) terms, guidelines and scoring sheet for cattle .*

QBA Terms:

| - Active | - Playful |
| --- | --- |
| - Relaxed | - Positively Occupied |
| - Fearful | - Lively |
| - Agitated | - Inquisitive |
| - Calm | - Irritable |
| - Content | - Uneasy |
| - Indifferent | - Sociable |
| - Frustrated | - Apathetic |
| - Friendly | - Happy |
| - Bored | - Distressed |

Classification:

Continuous scales for all body language parameters from minimum to maximum. The 20 descriptors are scored using the visual analogue scale (VAS) following observation. Each VAS is defined by its left ‘minimum’ and right ‘maximum’ point. ‘Minimum’ = the expressive quality indicated by the term is entirely absent. ‘Maximum’ = the expressive quality is dominant in observed animals. It is possible to give more than one term a maximum score, e.g., animals could be both entirely calm and content. To score each term, a line is drawn across a 125 mm scale at the appropriate point. The measure for that term is the distance in millimetres from the minimum point to the point where the line crosses the scale. Terms cannot be skipped. When scoring terms that start with a negative pre-fix, such as unsure or uncomfortable, as the score gets higher, the meaning of the score gets more negative, not more positive.

QBA Scoring Sheet:

Supplementary 2: Distribution of Qualitative Behaviour Analysis (QBA) and ankle-mounted accelerometer-based sensor variables correlated with QBA-PC1 (QBA derived Principal Component 1) for 107 cows from 4 herds. The first 10 terms correlated positively, and the following 8 terms correlated negatively with QBA-PC1.


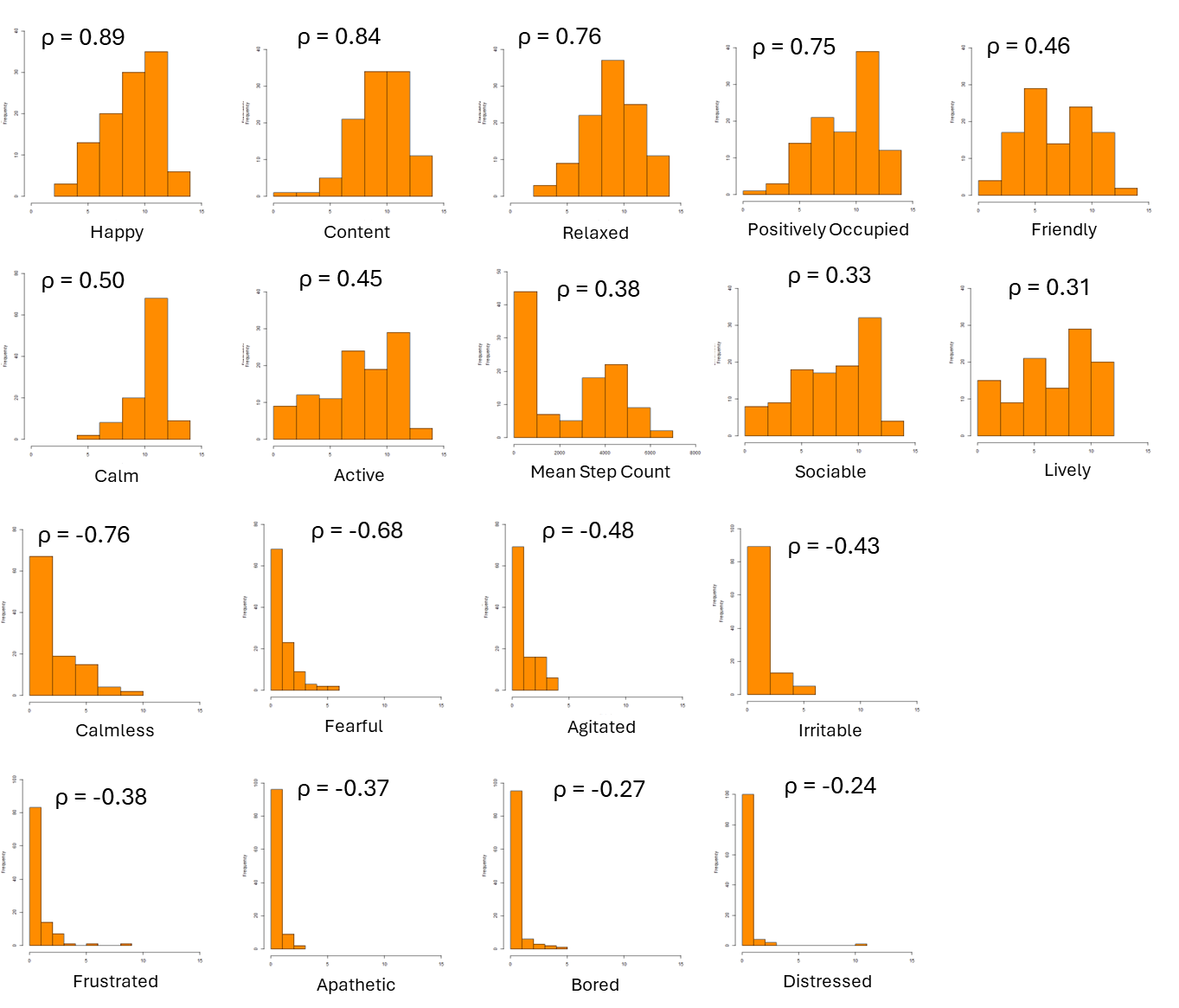

Supplement: Supplementary file 1 — Supplementary Material 1. [file 44363_2025_18_MOESM1_ESM.docx]
